# Submandibular fossa depth in relation to the mandibular canal and alveolar crest: a retrospective cross-sectional CBCT study analyzing side-specific and sex-based variations

**DOI:** 10.3389/froh.2026.1804700

**Published:** 2026-09-11

**Authors:** Nasser Raqe Alqhtani, Abdullah Saad Alqahtani, Abdulaziz Maree Alqahtani, Khalid Fahad Al-Harbi, Mohammed Abdullah Alshehri, Khalid Ayidh Alqahtani, Adel Alenazi, Fawaz Alqahtani, Mahmud Uz Zaman

**Affiliations:** 1 Department of Oral and Maxillofacial Surgery and Diagnostic Sciences, College of Dentistry, Prince Sattam Bin Abdulaziz University, Al-kharj, Saudi Arabia; 2 Department of Preventive Dental Sciences, College of Dentistry, Prince Sattam Bin Abdulaziz University, Al-kharj, Saudi Arabia; 3 General Dental Practitioner, Riyadh, Saudi Arabia; 4 Department of Prosthetic Dental Sciences, College of Dentistry, Prince Sattam Bin Abdulaziz University, Al-kharj, Saudi Arabia

**Keywords:** alveolar process, cone-beam computed tomography, dental implant, mandible, mandibular canal

## Abstract

An accurate preoperative evaluation of mandibular anatomy is essential before placing dental implants safely in the posterior region, where the submandibular fossa and proximity to the inferior alveolar nerve present serious risks. A precise preoperative evaluation of the mandibular anatomy is essential. This retrospective cross-sectional study evaluated the depth of SF using cone beam computed tomography (CBCT) and its correlation to the mandibular canal (MC) and alveolar crest (AC) in 200 adults (116 males, 84 females). Measurements were performed using the I-CAT Vision software and Intraclass Correlation Coefficient was used for the confirmation of intra- and interobserver reliability. Statistical analyses included Pearson correlations, t-tests, linear mixed-effects models (to account for bilateral correlations), multivariable regression and Benjamini-Hochberg false discovery rate correction. When controlling for within-subject correlation, sex differences remained significant on the right (*β* = 0.24, *p* = 0.009) but not the left (*p* = 0.081). Right side showed a weak negative correlation between SF depth and MC distance (r = −0.189, *p* = 0.007, r^2^ = 0.036), but this was not observed on the left side (*p* = 0.404). A lower MC position on the right was associated with higher SF depth (*p* = 0.027, q = 0.089). No relation with AC height. Despite sex- and side-related differences, the effect size is very weak (r = −0.189) and thus not suitable for prediction. Fossa depth was not a predictor of alveolar crest height, nor did it replace MC position evaluation. Sex and side specific anatomical risk varies, thus quadrant specific evaluation is necessary. These findings do not support standardized planning or SF depth alone as a clinical predictor. Instead, direct, independent CBCT measurements of both SF depth and MC distance at each implant site are necessary. Still, accurate 3D visualization of the SF–MC relationship is important to reduce the risk of lingual perforation and nerve injury. However, this visualization should be based on direct measurements rather than inferred correlations.

## Introduction

When planning dental implants in the lower posterior segment, it's crucial to consider not just the position of the implants but also how close they are to important nearby structures and the shape of the jawbone itself (1). Particularly, the posterior segment contains some vital anatomical landmarks—like the inferior alveolar nerve and the submandibular gland fossa—while important arteries such as the submental and sublingual pass through this area (2). The submandibular salivary gland presses against the inner side of the lower jaw, creating a natural hollow known as the submandibular fossa. This hollow typically extends from just behind the mental foramen below the mylohyoid line and can reach as far back as the lower wisdom teeth (3). Despite the fact the submandibular fossa and the mandibular canal are usually looked at separately in preoperative imaging, their spatial relationship to each other and to the alveolar crest is not often looked at in an integrated way. This is a missed clinical opportunity because placing an implant in the back of the mandible requires thinking about all three factors at the same time: the depth of the fossa (which could cause lingual perforation), the position of the inferior alveolar canal (which could cause nerve damage), and the available bone height from the alveolar crest.

Dental implants are a reliable and long-lasting way to restore missing teeth but placing them in the posterior segment of the lower jaw takes extra care. Dentists need to closely check important anatomical structures like the mandibular canal and the submandibular fossa before starting the procedure to avoid complications (4). Before implant placement, it is essential to thoroughly evaluate the mandibular canal, which houses the inferior alveolar nerve. Insufficient space between the implant and the nerve may lead to nerve damage, resulting in sensory changes or permanent numbness (5). Incorrect placement in these regions can also lead to lingual plate perforation and excessive bleeding (4). Detailed preoperative planning is key to keeping patients safe and achieving the best possible results (5).

In order determine the quantity and quality of bone at the implant site as well as the distance from important structures, radiographs are crucial for implant planning (6). Panoramic and periapical radiographs only display a limited amount of useful information due to their two-dimensional view. For dental implant planning, cone beam computed tomography (CBCT) is thought to be the best imaging technique (4). Compared to conventional two-dimensional radiography, it offers a more precise assessment of the depth and shape of the submandibular fossa as well as the location of the mandibular canal with respect to the implant site (7). The growing use of CBCT in clinical practice shows how important it is for improving surgical accuracy and patient safety. Because CBCT can make detailed, three-dimensional images, doctors often carefully look at important anatomical details and change how they put in implants to fit each patient's anatomy. This personalized approach to implant planning not only reduces the risk of complications but also improves long-term treatment outcomes for patients (7). The concavity of the fossa varies among individuals, with deeper fossae significantly increasing the risk of perforation during implant placement, which can result in serious consequences including hemorrhage or obstruction of the airway (8).

The anatomical features of the posterior mandibular area have been the subject of numerous investigations. Multiple research studies were done to calculate the distance of the submandibular fossa from the alveolar crest and its depth (6, 9–12) and Mandibular canal visualization (13–16) Nevertheless, these studies investigated the significance of these anatomical structures separately rather than correlating the anatomical findings with each other. It is important to look at each structure on its own, but knowing how they are related—like if a deeper submandibular fossa is more likely to be found in the same areas where the inferior alveolar canal is located more lingually or where the alveolar crest height is lower—could give a more complete picture of the patient's risk.

From a clinical perspective, understanding these correlations would enable the surgeon to anticipate multiple simultaneous risks instead of verifying them individually. If a deep submandibular fossa is statistically linked to a canal that is positioned more coronally or has more lingual decortication, then the presence of one feature should make you suspicious of the others right away. This could change how implants are planned. Instead of just avoiding the fossa or the nerve separately, the surgeon might move the implant buccally, choose a shorter implant, or skip the segment altogether when there are multiple bad features that happen at the same time.

Considering the recognized anatomical variability in the posterior mandible documented in the literature, it is not feasible to presume consistent or solid connections among these structures in advance. Therefore, this study sought to investigate the existence of significant correlations among the deepest point of the submandibular fossa, the distance from the alveolar crest, and the position of the inferior alveolar canal decortication (i.e., the point where the canal wall is thinnest or most lingually positioned), as well as to ascertain whether these relationships vary between males and females and between the right and left sides. Due to the known anatomical variability of the posterior mandible, we did not assume any strong or consistent correlations beforehand. Therefore, we hypothesized that the correlations between submandibular fossa depth, mandibular canal distance and alveolar crest height would be weak to moderate at best, and that side-specific and sex-based differences might exist. This exploratory study was designed to test these hypotheses. The aim of this study is to determine the deepest point of the submandibular fossa and to examine its correlation with the inferior alveolar canal decortication and the distance to the alveolar crest, thereby establishing a clinically relevant framework for preoperative risk assessment in posterior mandibular implant surgery.

## Materials and methods

### Study design

A retrospective cross-sectional analysis of CBCT datasets from patients who visited the College of Dentistry, Prince Sattam Bin Abdulaziz University, Al-Kharj, Saudi Arabia, for implant placement between March 2024 and December 2024. The study protocol adhered to the STROBE statement guidelines for cross-sectional studies and was conducted in accordance with the ethical principles of the *Declaration of Helsinki* (2013 revision). Ethical approval was obtained from the Institutional Review Board of Prince Sattam Bin Abdulaziz University (Approval Number: PSAU2020005).

### Sample sélection

CBCT based scans from 270 individuals were assessed, based on inclusion and exclusion criteria, 70 CBCT based scans were excluded, in total of 200 CBCT based scans (116 male, 84 female) with a total of 400 sided included in the present study, all data selected from the database of the Division of Oral and Maxillofacial Radiology after application of the following inclusion and exclusion criteria

- Inclusion criteria
  1. Healthy individuals without any oro-systematic disease.
  2. Adult individuals aged 18 years or older.
  3. All 3 or at least 2 lower molars present.
  4. CBCT-based dental records.
- Exclusion criteria:
  1. Presence of any systemic bone diseases.
  2. Patients with previously orthodontic treatment.
  3. Patients with previously implant treated or bone graft
  4. History of trauma

### Image acquisition

All the CBCT scans were taken using a CS 9300 3D apparatus (CARESTREAM/GERMANY) and subsequently evaluated by the CS 3DIMAGINGDental software. The CBCT images were acquired using specific parameters, i.e., 10 mA (current), 75 kVp (voltage), 0.2 mm (voxel size), 16 * 9-12 field of view (FOV).

### Image analysis

Cone beam computed tomography (CBCT) cross-sectional reconstructions were used for all measurements. The posterior mandible from the mental foramen to the retromolar region was the area of interest, with particular attention concentrated on the first and second molar regions where the submandibular fossa (SF) is most prominent.

#### Identification of the deepest point of the submandibular fossa

Cross-sectional images demonstrated that the deepest point of the SF was the point of maximum lingual concavity of the mandibular lingual plate. To ensure reproducibility, the deepest point was identified as the position where a line drawn perpendicularly to the lingual cortical plate exhibited the maximum perpendicular distance from a reference line linking the most superior and inferior points of the lingual concavity (refer to measurement method below). Before the study, the two examiners were calibrated in 20 CBCT scans not included in the final sample. Inter-examiner agreement was assessed at calibration and differences >0.3 mm were discussed until consensus was achieved. The standardized measurement protocol was described in a written manual with annotated reference images. Two independent examiners identified this point and disagreements were resolved by either a third examiner (senior oral radiologist with >10 years of experience) or unanimous consent. The inter-examiner ICC was 0.959 (right) and 0.970 (left) for the deepest point identification indicating excellent agreement.

#### Measurement of submandibular fossa depth

**Figure 1:**
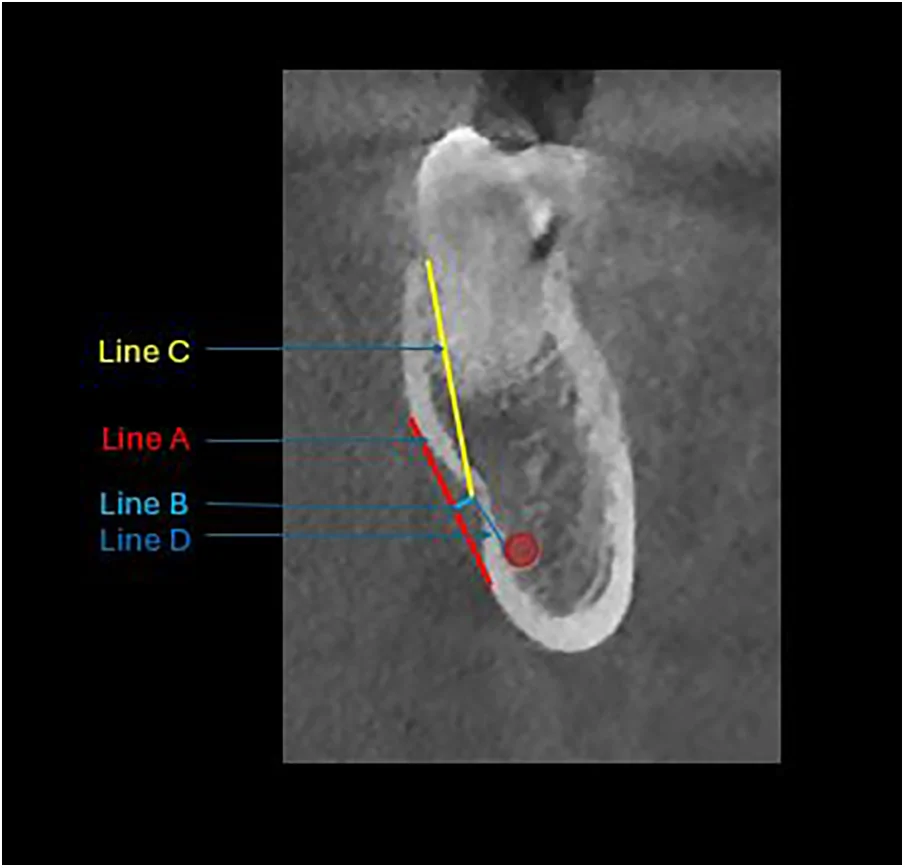
Submandibular fossa, distance from alveolar crest and relationship between mandibular canal and submandibular fossa. Line A (yellow): Reference line connecting superior and inferior points of lingual concavity. Line B (red): Submandibular fossa (SF) depth (perpendicular distance from deepest point to Line A; shown as 3.0 mm). Line C (blue): Alveolar crest (AC)-to-fossa distance (shown as 17.2 mm). Line D (green): Mandibular canal (MC)-to-fossa distance (shortest distance from most lingual canal point to deepest fossa point; shown as 1.3 mm). SF, submandibular fossa; MC, mandibular canal (inferior alveolar nerve canal); AC, alveolar crest; CBCT, cone beam computed tomography. Measurements in millimeters using I-CAT Vision software.

A line (Line A) was drawn between the highest and lowest points of the lingual concavity of the submandibular fossa. The highest point was the top of the lingual concavity, just below the alveolar crest. The lowest point was the bottom of the concavity, where the lingual plate starts to turn outward toward the bottom of the mandible. A second line (Line B) was drawn from the deepest part of the concavity straight down to Line A. The length of Line B (in millimeters) was used to measure the depth of the submandibular fossa. (See Figure 1).

#### Distance from alveolar crest to the deepest point of the submandibular fossa

The alveolar crest reference point was determined as the highest point of the alveolar crest in the cross-sectional image, specifically measured at the most coronal aspect of the ridge's lingual crestal point. A straight line (Line C) was drawn from the highest point of the lingual alveolar crest to the deepest part of the submandibular fossa (as shown above). This distance was measured in millimeters and shows the vertical and oblique distance from the crest to the deepest part of the fossa. Figure 1.

#### Measurement of distance from the inferior alveolar nerve canal to the deepest point of the submandibular fossa

The mandibular canal (inferior alveolar nerve canal) was observed as a radiolucent band with a thin radiopaque cortical line on the same cross-sectional image that was used to determine the deepest point of the SF. The reference point on the mandibular canal was designated as the most lingual point of the canal wall (lingual decortication point), as this indicates the nearest proximity of the canal to the submandibular fossa and the lingual plate. If the canal was not clearly corticated, the center of the canal was used as an alternative reference point, which was found by finding the middle point between the upper and lower borders and the buccal and lingual borders. A straight line (Line D) was drawn from the canal reference plane to the deepest point of the SF to the chosen reference point on the mandibular canal, It was measured in millimeters as the shortest straight-line distance between the two structures. Figure 1.

#### Classification of the mandibular canal position relative to the submandibular fossa

We examined a single cross-sectional image that revealed the deepest point of the submandibular fossa and classified the spatial relationship between the mandibular canal and that point into three groups: superior, parallel, and inferior. If the relationship varied between adjacent slices, the classification from the slice with the deepest fossa was used for analysis. The classification was defined as the position of the most lingual point of the mandibular canal relative to the horizontal line through the deepest point of the fossa: ’superior’ if the canal was above the line, ‘parallel’ if the canal was at approximately the same level (±0.5 mm), and ‘inferior’ if the canal was below the line. All the measurements were done using I-CAT Vision software (Imaging Science International, Hatfield, PA, USA). After interval of 2 weeks, the measurements in 16 patients were re-evaluated for intra-observer reliability and all measurements by using millimeter unit.

#### Reproducibility

Two independent examiners took all of the measurements twice, with at least two weeks between each session. We calculated the intraclass correlation coefficients (ICC) for continuous measurements, and for categorical classifications (superior/parallel/inferior), we calculated Cohen's kappa. An ICC or kappa value of 0.80 or higher was seen as a satisfactory level of agreement.

### Statistical analyses

1. Sample size

Initially a pilot study was conducted on 16 patients to calculate the number of samples needed for this study. The pilot study demonstrated that a sample size of 200 participants is sufficient to detect a clinically significant mean difference of 0.5 mm in submandibular fossa depth between groups (e.g., males and females or right and left sides), with a standard deviation of 0.5 mm, assuming 80% power and a two-sided significance level of *α* = 0.05. This threshold of 0.5 mm was chosen because it is larger than the CBCT voxel size (0.2 mm) and of a magnitude that may impact surgical decision-making (e.g., implant length or angulation). Sample size was calculated using NCSS 2004 and PASS 2000 program. The correlation analyses will have 80% power to detect a correlation coefficient of r = 0.20 (small effect size) at *α* = 0.05 with a sample size of 200. However, the study is not sufficiently powered to detect very small correlations (i.e., r < 0.15).

2. Inter-intra reader agreement

The data was wrangled, coded, and analyzed using the SPSS software (Armonk, NY, USA: IBM Corp version 25.0) and MedCalc (version 18.2.1, Ostend, Belgium). Inter-reader agreement and intra-reader agreement on the right and left deepest point of submandibular fossa measurements were tested using the Intraclass Correlation Coefficient (ICC) (ICC: <0.50, poor agreement; 0.50–0.75, moderate agreement; 0.75–0.90, good agreement; >0.90, very good agreement) and the Bland-Altman plot. Tables 1–3, Data were expressed using mean ± SD, (minimum and maximum). Independent and dependent t tests were to assess the difference in mean of quantitative variables between different categories. We also performed linear mixed-effects models with patient ID as a random effect to account for the non-independence of bilateral measurements (both right and left sides from the same patient). These models produced adjusted estimates with appropriate standard errors to account for within-subject correlation. Multivariable linear regression models were constructed to assess the independent effects of sex and side on fossa depth controlling for age and other possible confounders. Variables were chosen for inclusion based on clinical relevance and bivariate associations (*p* < 0.20). Standard diagnostic plots were used to check the assumptions of the model (linearity, homoscedasticity, normality of residuals). Pearson correlation was used to detect the relation between quantitative variables among studied cases. Given the number of comparisons, the Benjamini-Hochberg false discovery rate (FDR) correction was used to account for Type I error. FDR-adjusted *p*-values and q-values are reported. This correction is especially important for borderline significant findings, which should be interpreted with due caution. Statistical significance was considered when *p* < 0.05.

**Table 1:** Intraclass correlation coefficient between reader 1 and reader 2 (inter-reader reliability).

Variables	Intraclass correlation	95% CI (LL – UL)	F	p
Right deepest point of submandibular fossa	0.959	0.887–0.985	47.711	**<0** **.** 001*
Left deepest point of submandibular fossa	0.970	0.916–0.989	65.030	**<0**.001*

The asterisk (*) indicates statistical significance at the threshold of *p* < 0.05.

Bold *p*-values denote statistical significance (*p* < 0.05).

ICC values > 0.90 represent excellent inter-reader reliability.

**Table 2:** Intraclass correlation coefficient of intra-reader reliability (Reader 1).

Variables	Intraclass correlation	95% CI (LL – UL)	F	p
Right deepest point of submandibular fossa	0.989	0.968–0.996	173.663	**<0** **.** 001*
Left deepest point of submandibular fossa	0.985	0.956–0.995	128.614	**<0**.001*

The asterisk (*) indicates statistical significance at the threshold of *p* < 0.05.

Bold *p*-values denote statistical significance (*p* < 0.05).

ICC values > 0.90 represent excellent inter-reader reliability.

**Table 3:** Intraclass correlation coefficient of intra-reader reliability (Reader 2).

Variables	Intraclass correlation	95% CI (LL – UL)	F	p
Right deepest point of submandibular fossa	0.969	0.913–0.989	62.581	**<0** **.** 001*
Left deepest point of submandibular fossa	0.959	0.887–0.986	47.936	**<0**.001*

The asterisk (*) indicates statistical significance at the threshold of *p* < 0.05.

Bold *p*-values denote statistical significance (*p* < 0.05).

ICC values > 0.90 represent excellent inter-reader reliability.

## Results

A total of 200 patients were included in the study their mean age was (32.7 ± 9.2 years) ranged from 18 to 58 years old, from which 58.0% of them were males (*n* = 116) and 42.0% were females (*n* = 84). Given that of the retrospective cross-sectional design, it is not straightforward to establish causal relationships between anatomical variables; only associations can be expressed. Consequently, all statistically significant results, especially those with borderline *p*-values, must be interpreted with this limitation acknowledged and are optimally regarded as hypothesis-generating rather than conclusive. The sample size is big enough to find moderate correlations (r ≥ 0.20), but not big enough to find very weak correlations (r < 0.15). It is also important to think about how clinically important statistically significant results are. For example, a mean difference of less than 0.5 mm in fossa depth or canal distance may be statistically detectable, but it is unlikely to change surgical decision-making because CBCT voxel resolution is 0.2 mm and choosing an implant usually needs millimeter-level accuracy. The observed sex differences (0.24 mm right, 0.16 mm left) in the present study were below the pre-specified threshold of 0.5 mm to be of clinical relevance. These low magnitudes further support the conclusion that although there are anatomical differences between sexes, these are not of a magnitude to justify sex-specific surgical protocols.

**Figure 2:**
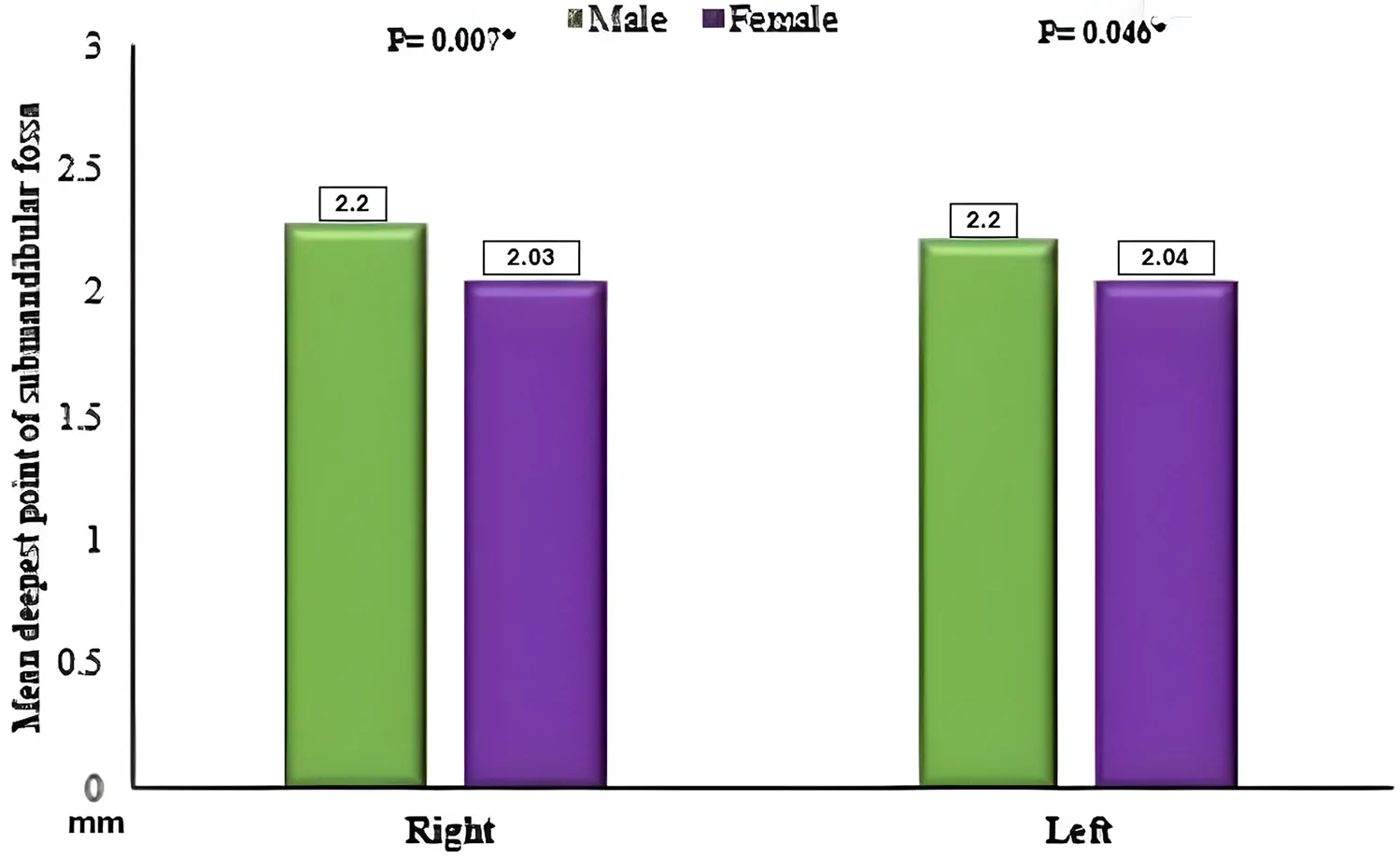
Right and left deepest point of submandibular fossa among studied patients. Grouped bar chart comparing mean submandibular fossa depth between males (*n* = 116) and females (*n* = 84) on right and left sides. Data shown as mean values (mm) with standard deviation error bars. *Y*-axis starts at 0.0 mm to accurately represent absolute differences and avoid visual overestimation. Statistical analysis (independent t-test with Benjamini-Hochberg FDR correction): Right side: males 2.27 ± 0.65 mm vs. females 2.03 ± 0.62 mm (*p* = 0.010, q = 0.032*). Left side: males 2.20 ± 0.53 mm vs. females 2.04 ± 0.51 mm (*p* = 0.046, q = 0.112). *Statistically significant after FDR correction (q < 0.05). Absolute sex differences are <0.25 mm, below the clinically meaningful threshold of 0.5 mm. SD, standard deviation; FDR, false discovery rate; mm, millimeters.

There was not statistically significant correlation between deepest point of submandibular fossa depth with age in both sides (Rt & Lt) as [(r = −0.08, *p* = 0.243) & (r = −0.072, *p* = 0.314) respectively]. Table 4. Right depth of deepest point of submandibular fossa was significantly higher among males than females (2.27 ± 0.65 among males compared to 2.03 ± 0.62 among females, *p* *=* *0.01, **q*** ***=*** ***0.032***. The left side of deepest point of submandibular fossa showed the same higher significant result among males compared to females (2.20 ± 0.53 vs. 2.04 ± 0.51, *p* *=* *0.046*); however, after Benjamini-Hochberg correction for multiple comparisons, this difference did not retain statistical significance (q = 0.112), indicating that this finding should be considered exploratory rather than confirmatory. Table 5, Figure 2**.** In the mixed-effects models that accounted for within-subject correlation, the sex-based difference in fossa depth remained statistically significant on the right side (*β* = 0.24, 95% CI: 0.06−0.42, *p* = 0.009), but the left side difference was attenuated and no longer significant (*β* = 0.16, 95% CI: −0.02 to 0.34, *p* = 0.081). In the multivariable linear regression model for right fossa depth, sex remained a significant independent predictor after adjusting for age (*β* = 0.22, 95% CI: 0.04−0.40, *p* = 0.017). Depth of fossa on either side was not significantly related to age. The model explained only 4.2% of the variation in fossa depth (adjusted R^2^ = 0.042), which supports the idea that sex and age are poor predictors of this anatomical feature.

**Table 4:** Correlation between deepest point of submandibular fossa depth with age in both sides (Rt & Lt).

Variable	Side	Test of significance	Age (years)
*Deepest point of submandibular fossa depth (n* *=* *200)*	Right	r	0.08
		p	0.243
	Left	r	0.072
		p	0.314

r; Pearson correlation.

**Table 5:** Compare between males vs. females as regards deepest point of submandibular fossa depth (Rt & Lt).

Variables	Male (*n* = 116)	Female (*n* = 84)	Test of significance (P)
* Right deepest point of submandibular fossa depth*	2.27 ± 0.65, (0.5–4.3)	2.03 ± 0.62, (0.5–3.2)	***t*** ***=*** ***−2.6,*** ***P*** ***=*** 0.01* ***q*** ***=*** ***0.032***
*Left deepest point of submandibular fossa depth*	2.20 ± 0.53, (1–3.6)	2.04 ± 0.51, (0.9–3.2)	***t*** ***=*** ***−2.01,*** ***P*** ***=*** 0 0.046* ***q*** ***=*** ***0.112***

The left side difference (*p* = 0.046) did not retain statistical significance after FDR correction (q = 0.112), indicating this finding should be considered exploratory rather than confirmatory.

Data described by Mean ± SD, (Min-Max).

Bold values denote statistical significance (*p* < 0.05) after Benjamini-Hochberg FDR correction.

*q* = FDR-adjusted *p*-value; values with *q* < 0.05 are considered statistically significant. The left side difference (*q* = 0.112) was not significant after correction.

t; Independent t test.

*Statistically significant.

**Figure 3:**
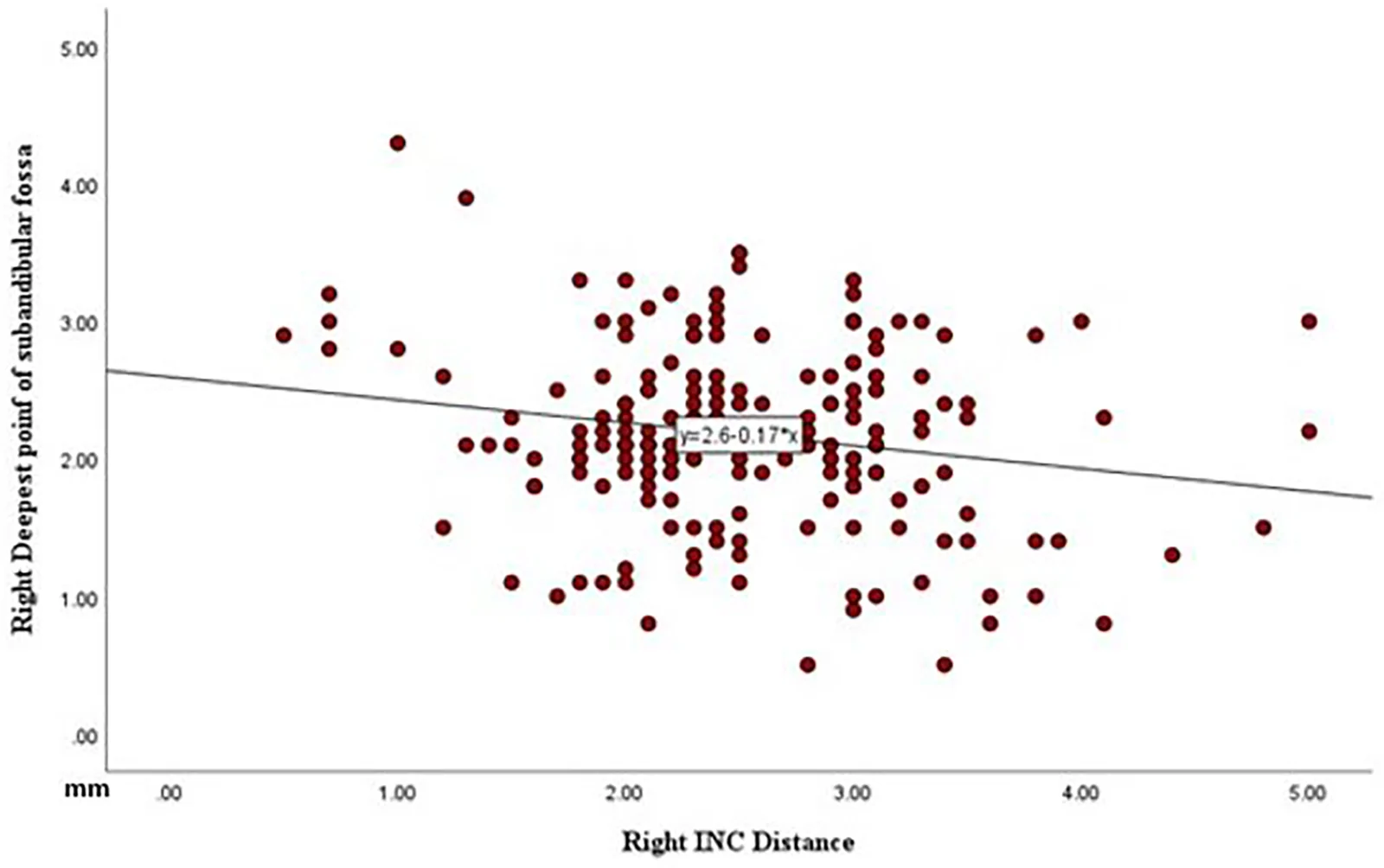
Correlation between right deepest point of submandibular fossa and right INC distance. Bland-Altman plot showing agreement between Reader 1 and Reader 2 for submandibular fossa depth measurements (*n* = 200 right sides). Solid line at Y = 0 represents perfect agreement; dashed lines represent 95% limits of agreement. ICC, 0.970 (95% CI: 0.916–0.989, *p* < 0.001), indicating excellent inter-reader reliability with no systematic bias. ICC, Intraclass Correlation Coefficient; CI, Confidence Interval; LoA, Limits of Agreement; mm, millimeters.

In all patients (regardless of the gender), both right and left deepest point of submandibular fossa were nearly comparable with no significant difference between them as the mean right deepest point was 2.17 ± 0.65, while the mean left deepest point was 2.13 ± 0.52), Table 6. Right side of deepest point of submandibular fossa showed a significant negative weak correlation with the right INC distance (r = −0.189, **95% CI: −0.322 to −0.051,** *p* = 0.007, **q** **=** **0.028**), Figure 3, while the left side showed a non-significant negative weak correlation with the left INC position.

**Table 6:** Compare between deepest point of submandibular fossa depth (Rt Vs Lt).

Variables	Rt deepest point of submandibular fossa depth (*n* = 200)	Lt deepest point of submandibular fossa depth (*n* = 200)	Test of significance (P)
*Mean* *±* *SD, (Min-Max)*	*2.17* *±* *0.65, (0.5–4.3)*	*2.13* *±* *0.52, (0.9–3.6)*	*t* *=* *1.02,* *P* *=* *0 0.3*

Data described by Mean ± SD, (Min-Max).

t; Dependent t test.

*Statistically significant.

**Figure 4:**
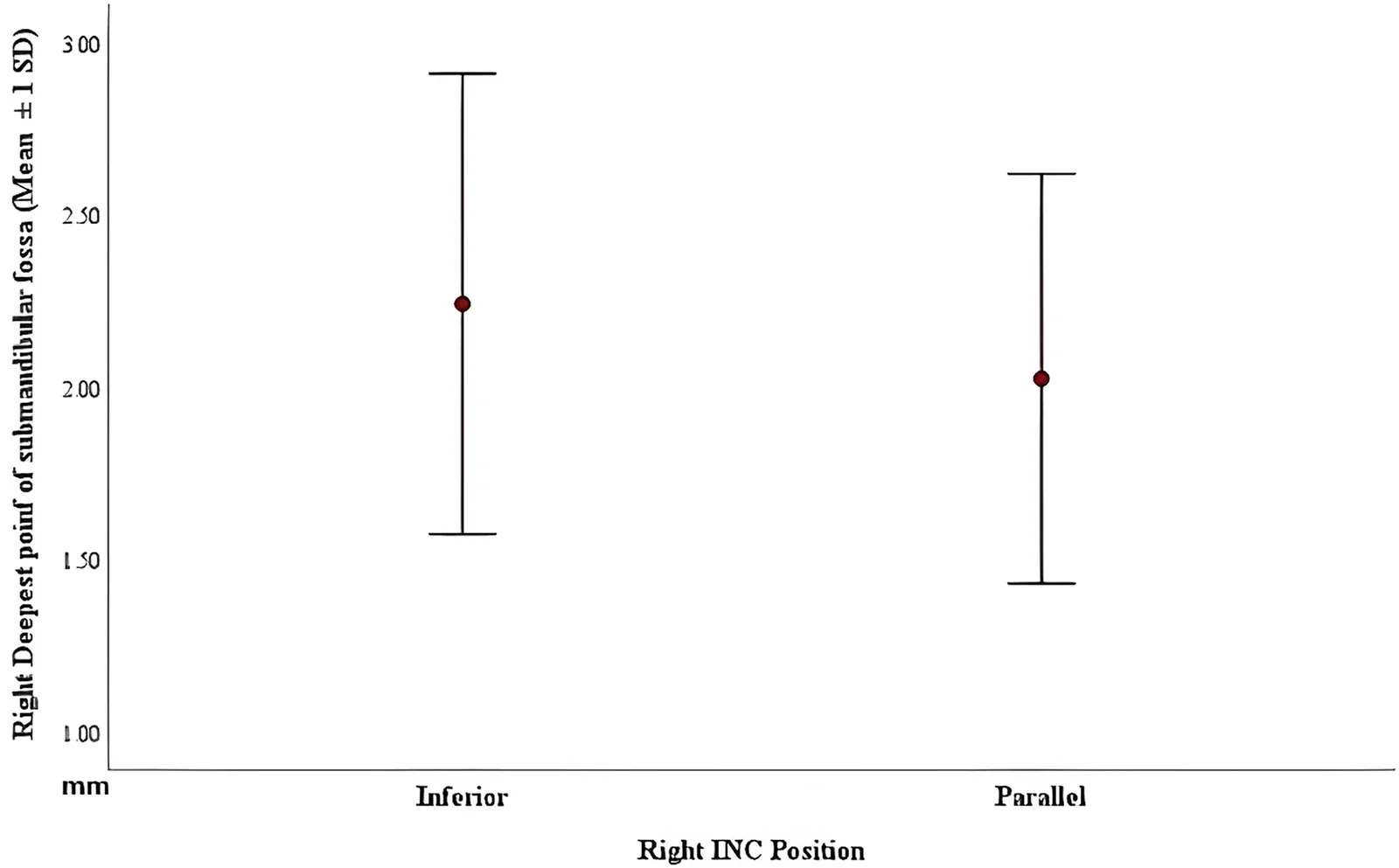
Right deepest point of submandibular fossa in relation to INC position. Bland-Altman plot showing agreement between Reader 1 and Reader 2 for right submandibular fossa depth measurements (*n* = 200 right sides). Solid line at Y = 0 represents perfect agreement; dashed lines represent 95% limits of agreement. ICC = 0.959 (95% CI: 0.887–0.985, *p* < 0.001), indicating excellent inter-reader reliability with no systematic bias. ICC, Intraclass Correlation Coefficient; CI, Confidence Interval; LoA, Limits of Agreement; mm, millimeters.

On the other hand, both right and left sides showed positive weak correlation with distance from alveolar crest, however this correlation was not statistically significant (r = 0.079 and 0.082, *p* = 0.27 and 0.25, respectively), The negative correlation on the right side (r = −0.189) is statistically significant but weak, only accounting for 3.6% of the variance (r^2^ = 0.036). The correlation is weak even at the upper bound as indicated by the 95% confidence interval (−0.322 to −0.051). This shows that fossa depth isn't a good way to guess where the mandibular canal is. The correlation existed solely on the right side; no significant correlation was detected on the left side (*p* = 0.404) Table 7. The right deepest point is significantly higher in inferior relation with right INC position than the parallel relation (2.24 ± 0.067 in inferior relation compared to 2.02 ± 0.59 in parallel relation, *p* = 0.027) However, this association was not statistically significant after FDR correction (q = 0.089). Therefore, this finding should be considered as hypothesis generating rather than definitive. Table 8, Figure 4. On contrary, the left deepest point was nearly equal in parallel and inferior relation of left INC position with no significant difference (2.12 ± 0.49 vs. 2.14 ± 0.54, *p* = 0.78), Table 9.

**Table 7:** Correlation between deepest point of submandibular fossa depth with distance from alveolar crest & INC distance in both sides (Rt & Lt).

Variables	Side	Test of significance	Distance from alveolar crest	INC distance
*Deepest point of submandibular fossa depth (n* *=* *200)*	Right	r	0.079	−0.189
		p	0.267	0.007* **95% CI: −0.322 to −0.051** **q** **=** **0.028**
	Left	r	0.082	−0.060
		p	0.248	0.404

Bold *p*-values and correlation coefficients denote statistical significance at *p* < 0.05 (and *q* < 0.05 after FDR correction).

r, Pearson correlation coefficient; CI, confidence interval; INC, inferior alveolar nerve canal.

The right side correlation (r = -0.189, p = 0.007, q = 0.028) is statistically significant but weak (r² = 0.036), indicating no clinical predictive value.

*Statistically Significant (*p* < 0.05).

**Table 8:** Relation between Rt deepest point of submandibular fossa depth with *INC position.*

Variables	INC position		Test of significance (P)
	Parallel relation (no. = 65)	Inferior relation (no. = 135)	
* Rt deepest point of submandibular fossa depth*	*2.02* *±* *0.59, (0.5–3.5)*	*2.24* *±* *0.67, (0.5–4.3)*	***t*** ***=*** ***−2.2,*** ***P*** ***=*** 0.027* ***q*** ***=*** ***0.089***

Data described by Mean ± SD, (Min-Max).

t; Independent t test.

Bold *p*-value denotes statistical significance at *p* < 0.05 prior to multiple comparison correction.

INC, inferior alveolar nerve canal; *q* = FDR-adjusted *p*-value. Although the difference was significant before correction (*p* = 0.027), it did not remain significant after FDR correction (*q* = 0.089), and thus should be interpreted with caution as hypothesis-generating.

*Statistically Significant (*p* < 0.05).

**Table 9:** Relation between Lt deepest point of submandibular fossa depth with *INC position.*

Variables	INC position		Test of significance (P)
	Parallel relation (no. = 53)	Inferior relation (no. = 147)	
* Lt deepest point of submandibular fossa depth*	*2.12* *±* *0.49, (1.2–3.6)*	*2.14* *±* *0.54, (0.9–3.2)*	*t* *=* *−0.28,* *P* *=* *0.78*

Data described by Mean ± SD, (Min-Max).

t; Independent t test.

## Discussion

Dentists have many alternatives for evaluating the jaw before implant therapy. Several studies document cases of lingual cortical perforation during attempted implant placement (11, 17–20) Notably, these studies did not indicate the standard use of CT imaging as a preventative measure. Panoramic radiographs are insufficient for implant planning due to their inherent limitations. While they allow for horizontal measurements and basic evaluation, their diagnostic value is compromised by image distortion, variable magnification, and the inability to provide three-dimensional visualization (15, 21). That's why nowadays preoperative 3D imaging is essential, as traditional assessment methods like palpation, casts, and conventional radiography have significant limitations in fully evaluating jaw health (22). In the last decade, three-dimensional imaging with cone-beam computed tomography (CBCT) has become the new standard, largely replacing conventional radiographic methods (23). Since sectional tomographic imaging offers a precise three-dimensional assessment, it is preferred over conventional two-dimensional radiographs (24). Cone-beam computed tomography (CBCT) is preferred over conventional CT in dental practice for a number of important reasons, including lower radiation doses, higher image resolution, and cost-effectiveness (10, 25). This enables precise measurement of the vertical bone size, buccolingual bone width, and implant angle—all of which are essential for implant success (24). Important anatomical features like the inferior alveolar canal and submandibular fossa make implant placement in the posterior mandible especially difficult. A lack of preoperative assessment increases the risk of serious consequences like hemorrhage, nerve damage, or lingual plate perforation (17, 26). These posterior concavities are formed by the pressure exerted by the adjacent submandibular salivary gland, which rests within the submandibular gland fossa (27). Lingual cortical perforation during implant placement is a frequent and significant risk, and the submandibular fossa is a common anatomical feature (10, 12, 18, 19, 28). Implants must be positioned appropriately to guarantee that occlusal forces are directed along the long axis of the ridge, which occasionally necessitates a tilted placement (29). The chance of this perforation during insertion is greatly increased by prominent fossa. CBCT precisely determines the submandibular gland fossa's depth and offers a detailed view of the mandibular structure (12, 30). The American Academy of Oral and Maxillofacial Radiology recommend using cross-sectional imaging to evaluate all potential implant sites (21, 22). Precise evaluation of alveolar bone type, dimensions, and the precise location of the mandibular canal is necessary for successful implant surgery (31). A thorough evaluation of the intended implant site in the posterior mandible is crucial due to the prevalence of deep lingual concavities (greater than 2 mm) in a variety of population groups (32).

Since the mandible grows significantly during puberty, mainly between the ages of 11 and 17, this study examined CBCT scans from patients 18 years of age and older (33). Males had bilaterally significantly deeper submandibular fossae than females, but there was no overall significant difference in depth between the left and right side. The distance to the mandibular canal and fossa depth had a significant negative correlation on the right side (r = −0.189, *p* = 0.007), but not on the left (*p* = 0.404). The statistically significant negative correlation between submandibular fossa depth and mandibular canal distance on the right side calls for careful interpretation. This very weak correlation (r^2^ = 0.036) only explains 3.6% of the variance, which means that fossa depth is not a good way to find the canal. The correlation is weak even at the upper bound as indicated by the 95% confidence interval (−0.322 to −0.051). This result does not clinically endorse the utilization of fossa depth as a surrogate for assessing canal distance. Instead, it highlights how important it is to directly and independently measure both anatomical structures on CBCT for each implant site. Overanalyzing this weak link could lead to mistakes in the clinic. Future studies should look for stronger predictors or models that use more than one variable. Alveolar crest distance and fossa depths had weak, nonsignificant positive correlations. Additionally, fossa depth was significantly higher on the right side when the mandibular canal was positioned inferiorly as opposed to parallel (2.24 ± 0.067 mm vs. 2.02 ± 0.59 mm, *p* = 0.027); but after FDR correction this association was not statistically significant anymore (q = 0.089) this difference was not observed on the left (*p* = 0.78). These findings emphasize how crucial it is to take individual anatomy into account when planning implants.

It is important to distinguish between statistical association and clinically actionable findings. Although sex differences were statistically significant and the correlation on the right side was weak, these results do not support clinically useful predictions. The correlation coefficient of −0.189 accounts for only 3.6% of the variance, indicating that 96.4% of the variation in mandibular canal position is not explained by fossa depth alone. Thus, we emphasize that these anatomical variables need to be evaluated independently and directly on CBCT for each implant site. Fossa depth cannot be used as a proxy or predictor for canal position and should not be used as such.

Male patients had a much deeper mandibular fossa than female patients, indicating that the bone at this location was narrower and thinner in men. This result is consistent with previous research by Koushal et al. (2022) (1) and Parnia et al. (2010) (10). In the area of the mandibular first molar, however, two other studies show that the most common type for both genders is a submandibular fossa depth of less than 2 mm (8, 34). Differences in the populations studied and the research techniques employed may account for the disparities in the results. More specifically, the observed differences are likely due to differences in CBCT acquisition parameters (voxel size, FOV), measurement reference points and the specific molar region studied (first vs. second molar). Additionally, ethnic and racial differences in mandibular morphology are well documented and results from Asian, European and Middle Eastern populations may not be directly comparable. The current Saudi Arabian cohort may exhibit unique anatomical characteristics that differ from previously studied populations. Studies by Rajput BS et al. (2018) (35) and Levingston et al. (2025) (34) found substantial gender differences in the mandibular second molar region. A submandibular fossa depth of less than 2 mm was more common in females, while a depth of more than 2 mm was more common in males. More than 3 mm of depth was rare and almost exclusively seen in male patients (35). The current study did not find a statistically significant gender-based difference, in contrast to those findings. The average depths on the right side were 2.27 ± 0.65 mm for men and 2.03 ± 0.62 mm for women (*p* = 0.01). Males measured 2.20 ± 0.53 mm on the left side, while females measured 2.04 ± 0.51 mm (*p* = 0.046). These results are consistent with earlier studies showing that mandibular bone dimensions are generally greater in males (1, 9, 34). Underlying variations in skeletal morphology, hormone profiles, and bone density may be responsible for this sexual dimorphism. An intriguing finding from the Levingston et al. (2025) study was that females showed a deeper submandibular fossa on the left side than males in the second molar region. Additionally, in the first and second molar regions, the fossa depth was consistently deeper on the left side for both genders (34). These findings align with the findings of Ramaswamy et al. (2020), who observed a deeper submandibular fossa on the left side of the mandible (36). On the other hand, similar to our study, Yildiz et al. (2014) reported a deeper right side, highlighting how this anatomical feature can differ among various population groups (37). The depths of the right and left submandibular fossae in the entire study sample did not differ statistically significantly, indicating a general symmetry in mandibular anatomy.

Particularly on the right side, a significant negative correlation was discovered between a deeper submandibular fossa and a closer mandibular canal (r = −0.189, *p* = 0.007). On the left side, this link was absent (*p* = 0.404). These results are consistent with the findings published by de Bayrak, S. et al. (8) and Souza et al. (29), even though those studies didn't investigate differences in correlation strength between sides. The observed inverse correlation is expected because a narrower mandible is reflected in a deeper submandibular fossa, which shortens the distance between the mandibular canal and the cortical bone. According to Bayrak, S. et al., the submandibular fossa is deepest bilaterally around the second molar and progressively shallower mesially and distally, reaching its minimum depth at the second premolar (8). This result highlights the significance of careful planning in these anatomical scenarios by suggesting that a deeper, more concave fossa increases the risk of injuring the inferior alveolar nerve during an implant procedure. As a result, the risk of perforating the submandibular fossa is reduced when implants are positioned in the premolar region and progressively increases towards the more posterior molar area. The finding of a substantial negative correlation between submandibular fossa depth and mandibular canal distance exclusively on the right side, and not on the left, necessitates careful interpretation. There are a few possible reasons for this asymmetry, but the current data can't confirm any of them.

First, it is well known that the masticatory system is functionally asymmetrical, and that most people have a preferred side for chewing. Theoretically, increased and more frequent mechanical loading on the dominant side could affect regional bone remodeling, resulting in slight changes in the shape of the mandible, such as the depth of the submandibular fossa and the location of the mandibular canal. Second, the anatomy of the inferior alveolar canal may differ between sides; the canal is not always perfectly symmetrical, and its lingual decortication may be more pronounced on one side due to developmental or genetic factors. Third, the observed asymmetry might just be a fortunate finding resulting from sampling variability or a Type I error, especially considering the weak correlation (r = −0.189) and the occurrence of multiple statistical comparisons. Fourth, variations in the positioning of the submandibular salivary gland or in the pattern of fossa formation between the right and left sides may play a role, although these differences have not been thoroughly marked in the literature.

The fundamental cause of this side-specific correlation remains unreliable in the present study. The current study was not intended to assess lateral asymmetries, and the sample size was not specifically optimized for side-to-side comparisons. Consequently, this finding ought to be regarded as hypothesis-generating rather than conclusive. Subsequent research involving larger, more representative samples and focused examinations of bilateral asymmetry is essential to ascertain whether this side-specific correlation signifies a genuine biological phenomenon or merely a statistical artifact. Until such evidence is available, clinicians must not presume that anatomical relationships observed on one side of the mandible consistently predict those on the contralateral side. Furthermore, preoperative CBCT assessment should be conducted separately for each proposed implant site, irrespective of side.

Our results show a different anatomical profile from those reported by Lascala et al. (2004) (38) and Vhatkar et al. (2019) (39), but they agree on a crucial clinical principle. Although Vhatkar reported a shallower range of fossa depths (0.5–3.70 mm) and Lascala reported a significantly deeper submandibular fossa (6 ± 2.6 mm) with a shorter associated alveolar bone height (4.2–11.9 mm), their study consistently recorded a greater vertical distance from the fossa to the alveolar crest (∼14.01–14.8 mm) (38). This disparity in absolute measurements is probably the result of different study populations or methodologies. But this comparison reveals a crucial consensus. Our analysis revealed only a weak, non-significant correlation between fossa depth and alveolar crest height, despite the large variation in fossa depth across these studies. This supports the conclusion that fossa depth is an independent and unreliable predictor of the available vertical bone superior to the mandibular canal and is consistent with the implication from Lascala's data (38), where a very deep fossa coexisted with a relatively short bone height. As a result, all three datasets highlight the same necessity for clinical practice: fossa depth alone cannot determine the risk of lingual perforation or the safety of implant placement; instead, a thorough 3D evaluation is required to measure the bone height above the inferior alveolar canal in each unique case.

A significant anatomical relationship, particularly on the right side, was found when the submandibular fossa depth was compared to the location of the inferior alveolar canal. The fossa was significantly deeper (2.24 ± 0.67 mm) when the canal ran more inferiorly than when it ran parallel (2.02 ± 0.59 mm) (*p* = 0.027); after FDR correction, this association was no longer statistically significant (q = 0.089). The left side did not exhibit this association (*p* = 0.78). A similar pattern was noted by Ramaswamy (2020), who found that the deepest point was typically below the canal in males and at the level of the canal in females (36). In contrast, Levingston et al. found that the deepest portion of the submandibular fossa, in relation to the mandibular canal, was more frequently located superior to the canal in females than in males on both sides of the mandibular first molar, although this trend was not statistically significant (34). This is consistent with the results of Haghanifar et al., who observed that in both sexes, the deepest fossa regions were above the canal (2). To determine safe implant placement and angulation, it is crucial to assess such individual anatomical variations during preoperative planning, as these findings demonstrate that the spatial relationship between these two structures can vary. Variations in demographic characteristics, such as age, ethnicity, and the specific location of the mandible being assessed.

Anatomical relationships seem to be consistent across most research projects, but demographic factors can cause variations that call for a customized approach to dental implant planning, according to a closer look at the data. Clinically, results of this study highlight the need for a complete preoperative CBCT evaluation. Before surgery, three parameters should be evaluated with a CBCT scan: (1) the depth of the submandibular fossa, (2) the distance between the fossa and the mandibular canal and (3) the height of the alveolar crest in relation to the canal. This triple assessment is supported by many studies that show that in the presence of a deep fossa (>2 mm) with a canal in close proximity (<2 mm) the risk of lingual cortical perforation and injury to the inferior alveolar nerve during implant placement is significantly increased (4,8,10,17-19,24,26,29). The perforation of the lingual cortex is a frequent and serious complication of implant placement and the submandibular fossa is a common anatomic feature (10,12,18,19,28). A prominent fossa greatly increases the chance of this perforation occurring during insertion. CBCT accurately determines the depth of the submandibular gland fossa and provides a detailed view of the mandibular structure (12,30). The American Academy of Oral and Maxillofacial Radiology recommend cross-sectional imaging for the evaluation of all potential implant sites (21,22).

In high-risk anatomical situations with a deep fossa (>2 mm) and a closely positioned canal (<2 mm), clinicians should consider changing their surgical approach by using shorter implants, shifting the implant axis buccally or avoiding the site altogether in lieu of alternative restorative options (24,29). This recommendation is consistent with the guidelines of the American Academy of Oral and Maxillofacial Radiology which recommend cross-sectional imaging evaluation of all potential implant sites (21,22). Special attention should be paid to the posterior mandible of male patients who showed significantly deeper fossae bilaterally. While the absolute difference is small (0.16–0.24 mm) and perhaps not worth sex-specific protocols, awareness of this anatomic trend should promote careful assessment of each individual. The surgeon should consider the use of shorter implants, buccal displacement of implant axis or staged bone augmentation procedures in cases of deep fossa (>3 mm) especially associated with inferiorly displaced canals. If both adverse features are present (deep fossa + close canal proximity) then the site should be avoided altogether and alternative restorative options considered.

Future studies should broaden their scope to include a larger population and consider longitudinal studies that look at how bone structure changes over time and how this affects dental implant success rates. More investigation into how sophisticated imaging techniques enhance physicians' diagnostic skills could help with this. Understanding the similarities and differences between different dental implant outcomes would be very beneficial to dentistry. Future studies should include these clinical factors for a more complete assessment of the implant location selection. Specifically, these findings require validation and extension through multicenter prospective studies across various ethnic populations. In addition, the use of multivariable predictive models incorporating bone density measurements (Hounsfield units), cortical thickness, and three-dimensional canal trajectory would enhance the risk stratification. Machine learning approaches such as random forests or gradient boosting machines may be able to detect nonlinear interactions among the anatomical variables that traditional regression models cannot. Such models trained on large multicenter datasets may ultimately be able to produce patient-specific risk scores for lingual perforation and nerve injury, thus allowing truly personalized implant planning. However, it is important to emphasize that any predictive model must be validated in independent external cohorts prior to clinical deployment.

Despite offering valuable anatomical data, this study has a number of limitations. Its focus on a particular Saudi population restricts the results' applicability to other ethnic groups, and its retrospective nature from a single center may result in selection bias. The retrospective cross-sectional design of this study further limits the strength of inference, only associations can be determined and not causal relationships. The sample size was based on the ability to detect a mean difference of 0.5 mm, which is a clinically meaningful threshold, although statistically feasible. A CBCT voxel resolution of 0.2 mm and the necessity of millimetre accuracy for surgical planning make a difference in fossa depth or canal distance of less than 0.5 mm unlikely to influence the choice of implant, e.g., length, diameter, or angulation. Thus, although some results were statistically significant, their clinical relevance may be questionable. In addition, CBCT, while providing superior structural detail, does not capture important patient-specific characteristics such as bone quality and density. Therefore, borderline significant results (e.g., sex-based difference on the left side, *p* = 0.046, deeper fossa with inferior canal position, *p* = 0.027) should be interpreted with caution as hypothesis-generating rather than confirmatory, especially after false discovery rate correction where these findings were no longer statistically significant (q = 0.112 and q = 0.089, respectively), requiring validation in prospective cohort studies. Several statistical comparisons were performed without adjustment for multiple testing increasing the risk of Type I error. Thus, the borderline results should be considered exploratory and hypothesis generating rather than definitive. Additionally, the weak correlation observed between fossa depth and mandibular canal distance (r = −0.189) limits any clinical predictive application, and future studies should focus on identifying stronger, more reliable anatomical predictors or multivariable models that incorporate bone density, cortical thickness, and canal position simultaneously. Measurements from both sides of the mandible were included from each patient. These observations are not statistically independent. We dealt with this by using mixed effects models, but this non-independence was not accounted for in the main analyses using t-tests and Pearson correlation, which is a methodological limitation. Future studies that incorporate these clinical factors would provide a more thorough evaluation for choosing appropriate implant locations.

## Conclusion

The findings reveal that the submandibular fossa has a lot of anatomical variation, with males having a much deeper fossa than females although the absolute difference is small (<0.25 mm) and of limited clinical consequence. A deeper fossa exhibits a weak yet statistically significant correlation with a shorter mandibular canal distance on the right side (r = −0.189); however, fossa depth alone cannot reliably predict canal position, necessitating direct measurement of both structures. It is crucial to note that fossa depth does not correlate with vertical alveolar bone height. The weak correlation (r2 = 0.036) suggests that fossa depth explains only 3.6% of the variability in canal position, highlighting the necessity to evaluate these anatomical factors independently and directly on CBCT for each implant site. Canal position must not be used as a proxy or predictor of fossa depth. This variable anatomy, while generally symmetrical, highlights the necessity for customized three-dimensional CBCT assessment instead of depending on panoramic radiographs or single-dimensional measurements. Regular evaluation of the fossa–canal relationship ought to be an integral aspect of implant planning in the posterior mandible.

## Data Availability

The original contributions presented in the study are included in the article/Supplementary Material, further inquiries can be directed to the corresponding author.
